# Adjuvant Chemotherapy Might Be Recommended to Patients With Positive Margin After Gastrectomy: A 20-Year Retrospective Analysis in a Single Center

**DOI:** 10.3389/fonc.2021.794032

**Published:** 2022-02-02

**Authors:** Xiaojie Zhang, Lulu Zhao, Penghui Niu, Tongbo Wang, Wanqing Wang, Chongyuan Sun, Zefeng Li, Yingtai Chen, Dongbing Zhao

**Affiliations:** Department of Pancreatic and Gastric Surgical Oncology, National Cancer Center/National Clinical Research for Cancer/Cancer Hospital, Chinese Academy of Medical Sciences and Peking Union Medical College, Beijing, China

**Keywords:** gastric cancer, positive margin, prognosis, adjuvant chemotherapy, gastrectomy

## Abstract

**Background:**

Margin positivity after gastric cancer resection is associated with poorer outcomes. However, the prognostic factors and the choice of postoperative adjuvant treatment of patients with positive margin (PM) after gastrectomy are still being debated.

**Methods:**

A single-center, retrospective analysis was conducted for patients with PM after gastrectomy from the China National Cancer Center Gastric Cancer Database (NCCGCDB) from 1998 to 2018. Univariate and multivariate Cox regression analyses were performed to identify prognostic factors of overall survival (OS) and recurrence-free survival (RFS).

**Results:**

A total of 449 patients were included in the study, including 192 (42.8%) in the proximal PM group (PPM), 205 (45.7%) in the distal PM group (DPM), and 52 (11.6%) in the bilateral PM group (BPM). The 3- and 5-year OS rates for the PM patients investigated were 47.5% and 39.3%, respectively, and the 3- and 5-year RFS rates were 60.0% and 53.6%, respectively. Multivariate Cox regression analysis proved total gastrectomy (hazard ratio (HR): 1.783, 95%CI: 1.133–2.805, *p* = 0.012), pT4 (HR: 5.264, 95%CI: 1.493–18.565, *p* = 0.01), pN2 (HR: 2.263, 95%CI: 1.164–4.397, *p* = 0.016), pN3 (HR: 2.327, 95%CI: 1.233–4.393, *p* = 0.009), and combined resection (HR: 1.952, 95%CI: 1.256–3.034, *p* = 0.003) to be independent risk factors of OS, and pT3 (HR: 9.257, 95%CI: 1.152–74.386, *p* = 0.036) and pT4 (HR: 11.361, 95%CI: 1.469–87.847, *p* = 0.020) to be independent risk factors for RFS. Adjuvant chemotherapy prolonged OS in the PPM group (*p* = 0.032) and prolonged RFS in the PPM group (*p* < 0.001) and the DPM group (*p* = 0.035) compared with surgery alone.

**Conclusions:**

Advanced pathologic stage was associated with poor prognosis, and postoperative adjuvant chemotherapy might be recommended in PM patients after gastrectomy. Still, further prospective trials are warranted to verify and support our conclusions.

## Introduction

Gastric cancer (GC) is the fifth most commonly diagnosed cancer and the third leading cause of cancer death worldwide, with more than 1 million new cases and nearly 800,000 deaths annually (1). Surgical resection is a potentially curative approach treatment for resectable GC, but recurrence and metastasis are still occurring at high rates (2). Resection with a negative margin (R0) was verified to be one of the most important prognostic factors for this aggressive tumor (3). Positive resection margin (PM) includes microscopic (R1) and macroscopic (R2) tumor cells visible on the resection margin (4). Regardless of improvements in surgical technique and intraoperative frozen section examination, the incidence of PM still reached 1.8%–5.1% for GC (5). In GC, multiple previous studies have demonstrated that PM represents an independent risk factor for poor prognosis and increased risk for recurrence (5–10). Due to the low incidence of PM, there is still a lack of adequate research on the clinical features and prognostic factors.

There is currently no consensus on the treatment strategy for the PM patients after the operation (11). The current National Comprehensive Cancer Network (NCCN) guidelines recommend the use of adjuvant concurrent chemoradiation (CCRT) after R1 resection, although the writers of the guideline acknowledge that this strategy has not been evaluated in prospective trials (12, 13). However, the choice of postoperative treatment in clinical practice still varies widely among many centers (11, 14, 15). A *post hoc* subgroup analysis showed that adjuvant CCRT improved survival time as compared with surgery alone, despite only 22 PM patients being enrolled (16). In addition, when comparing CCRT and chemotherapy, a National Cancer Database (NCDB) study showed that chemoradiotherapy was associated with improved overall survival (OS) time (14). However, this study did not assess the surgery alone group. Furthermore, limitations of the NCDB include selection bias, lack of clinically relevant endpoints like the cause of death, and disease-free survival (17). Paradoxically, a recent retrospective study with 69 PM patients did not find improved survival time with adjuvant treatment (18). Therefore, our study aims to examine the prognostic clinicopathological characteristics of GC with PM and investigate the impact of postoperative management strategies on local control and long-term survival.

## Methods

### Patients

All the study data were abstracted from the China National Cancer Center Gastric Cancer Database (NCCGCDB) from 1998 to 2018. A detailed description of the database has been previously published (19). All patients meeting the following criteria were eligible for inclusion in this study. The inclusion criteria included the following: i) adenocarcinoma of the stomach; ii) stage I to IV underwent gastrectomy; and iii) postoperative histopathology confirmed PM based on paraffin‐embedded tissue. The exclusion criteria were as follows: i) with the history of other malignant cancers; ii) death during the hospital stay or within 1 month after the operation; and iii) underwent endoscopic resection. Eventually, a total number of 449 patients fulfilled the criteria.

The main covariates include demographic characteristics, detailed preoperative clinical information, surgery-related information, postoperative pathologic results, and recurrence and metastasis. GC stages were classified according to the criteria of the American Joint Committee on Cancer (AJCC; 8th edition). According to the location of the positive resection margin, we divided all patients into three groups: bilateral PM group (BPM), proximal PM group (PPM), and distal PM group (DPM).

### Postoperative Chemotherapy

In total, 158 (35.2%) patients received 3–8 cycles of postoperative chemotherapy. Chemotherapy regimens consisted of the following: i) S-1/oxaliplatin (SOX, 30 patients); ii) capecitabine/oxaliplatin (XELOX, 24 patients); iii) fluorouracil/leucovorin/oxaliplatin/docetaxel (FLOT, 6 patients); iv) S-1/oxaliplatin/docetaxel (DOS, 13 patients); v) oxaliplatin/leucovorin/fluorouracil (FOLFOX, 7 patients); vi) cisplatin/paclitaxel (TP, 2 patients); vii) cisplatin/docetaxel (DP, 1 patients); viii) S-1 monotherapy and S-1/nab-paclitaxel (5 patients); ix) capecitabine (2 patients); x) cisplatin/5‐fluorouracil/docetaxel (DCF, 2 patients); xi) etoposide/doxorubicin/cisplatin (EAP, 2 patients); xii) cisplatin/epirubicin/tetrahydrofolate/fluorouracil (PELF, 3 patients); xiii) others (30 patients); and xiv) unknown (31 patients).

### Postoperative Chemoradiotherapy

A total number of 35 (7.8%) patients received chemoradiotherapy, which includes chemotherapy followed by radiotherapy (CRT) and CCRT alone. For CRT planning, chemotherapy regimens consisted of SOX and XELOX. The total radiotherapy dose ranged from 45 to 60 Gy. For CCRT planning, patients received intensity-modulated radiotherapy (IMRT) plus oral S-1 or capecitabine. IMRT was planned for patients with prescription doses of 40.04–53.76 Gy for planning target volume one and 43.2–54 Gy for planning target volume two.

### Follow-Up

The long-term follow-up was performed by outpatient clinical visits and telephone contact. The main outcomes were OS time and recurrence-free survival (RFS) time. We defined OS as the time from surgery to the time of the last follow-up or the time of death. RFS was defined as the time from the date of surgery until local recurrence or distant metastasis. If the patient is lost to follow-up, the follow-up time is censored. The last follow-up time was September 2020, and the duration follow-up time was 1~229 months, with a median follow‐up time of 23.3 months. A total of 120 patients were lost to follow-up, and the follow-up rate was 73.3%.

According to the site of the first recurrence, the recurrence patterns were divided into locoregional recurrence and distant recurrence. Locoregional recurrence was defined as tumor recurrence of resection margin, regional lymphatic vessels, anastomosis, or the tumor bed. Distant recurrence was defined as a tumor metastasis occurring in other organs, such as the liver, lungs, and peritoneal or non-regional lymph nodes (5, 18). Recurrences were established based on physical examinations, with imaging results basically. For patients with doubts about the diagnosis of recurrence, the recurrence site is confirmed by biopsy.

### Statistical Analysis

Statistical analysis was performed using the SPSS program version 22.0 for Windows (SPSS Inc., Chicago, IL, USA). All categorical data were displayed as frequencies and percentages, and continuous data were expressed as mean and SD. Survival analyses were performed using Cox proportional hazards regression analysis. Factors that were deemed of potential importance to identify independent risk factors on the univariate analysis (*p* < 0.1) were included in the multivariate analysis. Multivariable Cox proportional hazards regression was performed to adjust for confounders. Hazard ratios (HRs) with 95% CIs were obtained as a measurement of association. The Kaplan–Meier survival curve was plotted by use of a GraphPad Prism, version 8.0.2 (GraphPad Software, La Jolla, CA, USA). *p* < 0.05 was considered statistically significant.

## Results

### Demographics of the Patients

The clinicopathological characteristics of the study population are summarized in **Table 1**. A total of 449 patients were included in the study, including 192 (42.8%) in the PPM group, 205 (45.7%) in the DPM group, and 52 (11.6%) in the BPM group. Overall, 37 (8.2%) patients received R2 resection. The proportions of R2 resection in the three groups were similar. The median age (range) was 60 years (28–89 years), and the majority (72.6%) was male. Only 8.2% of patients received neoadjuvant treatment, and 4.7% were gastric stump carcinoma. In addition, 42 patients underwent gastrectomy for stage IV GC, and 2 patients received combined resection for radical purposes, while 40 patients received palliative resection due to the obstruction and bleeding. In all patients, 134 (29.8%) were positive in intraoperative frozen pathologic examination, and 71 (15.8%) were negative. In these 134 patients with positive in intraoperative frozen pathologic examination, the reasons for not achieving R0 by additional resection were as follows: 1) 9 patients were stage IV with palliative resection; 2) 14 patients received combined resection, and the extended radical gastrectomy was not suitable; and 3) 111 patients received the extend gastrectomy, but the postoperative pathologic examination showed positive surgical margins. From the pathologic features, most of the patients are T4 (75.5%), and 387 patients (86.2%) had regional lymph node metastasis. Overall, 112 patients (24.9%) underwent combined resections. The details are shown in **Supplementary Table 1**. In terms of postoperative treatment options, 52 (11.6%) people did not receive treatment, 158 (35.2%) received chemotherapy alone, 35 (7.8%) received chemoradiotherapy, and 15 (3.3%) received secondary surgical resection.

**Table 1:** Clinicopathological characteristics of the patients with positive margin after gastrectomy.

Characteristic	Total	Margin involved		
		Proximal	Distal	Both
	N = 449 (%)	N = 192 (%)	N = 205 (%)	N = 52 (%)
Age (mean ± SD)	58.9 ± 11.8	59.8 ± 11.4	58.4 ± 11.8	57.5 ± 14.4
BMI (mean ± SD)	23.2 ± 3.4	23.2 ± 3.6	23.3 ± 3.4	22.8 ± 3.1
Gender				
Male	326 (72.6)	136 (70.8)	151 (73.7)	39 (75.0)
Female	123 (27.4)	56 (29.2)	54 (26.3)	13 (25.0)
Tumor location				
Proximal	175 (39.0)	89 (46.4)	74 (36.1)	12 (23.1)
Distal	248 (55.2)	91 (47.4)	123 (60.0)	34 (65.4)
Middle	24 (5.3)	11 (5.7)	8 (3.9)	5 (9.6)
Unknown	2 (0.4)	1 (0.5)	0 (0.0)	1 (1.9)
Neoadjuvant therapy				
No	385 (85.7)	159 (82.8)	182 (88.8)	44 (84.6)
Yes	37 (8.2)	21 (10.9)	12 (5.9)	4 (7.7)
Unknown	27 (6.0)	12 (6.3)	11 (5.4)	4 (7.7)
Gastric stump carcinoma				
No	342 (76.2)	147 (76.6)	155 (75.6)	40 (76.9)
Yes	21 (4.7)	10 (5.2)	8 (3.9)	3 (5.8)
Unknown	86 (19.2)	35 (18.2)	42 (20.5)	9 (17.3)
Resection type				
PG*	183 (40.8)	85 (44.3)	81 (39.5)	17 (32.7)
DG*	143 (31.8)	38 (19.8)	88 (42.9)	17 (32.7)
TG*	79 (17.6)	48 (25.0)	20 (9.8)	11 (21.2)
Unknown	44 (9.8)	21 (10.9)	16 (7.8)	7 (13.5)
Surgical approach				
Open	370 (82.4)	160 (83.3)	171 (83.4)	39 (75.0)
Laproscope	79 (17.6)	32 (16.7)	34 (16.6)	13 (25.0)
Tumor size (pathology)				
<5 cm	89 (19.8)	39 (20.3)	42 (20.5)	8 (15.4)
≥5 cm	283 (63.0)	125 (65.1)	133 (64.9)	25 (48.1)
Unknown	77 (17.1)	28 (14.6)	30 (14.6)	19 (36.5)
Intraoperative frozen pathology				
Negative	71 (15.8)	32 (16.7)	33 (16.1)	6 (11.5)
Positive	134 (29.8)	60 (31.3)	61 (29.8)	13 (25.0)
None	244 (54.3)	100 (52.1)	111 (54.1)	33 (63.5)
Differentiation				
Well and moderate	55 (12.2)	29 (15.1)	21 (10.2)	5 (9.6)
Poor and undifferentiated	338 (75.3)	141 (73.4)	158 (77.1)	39 (75.0)
Unknown	56 (12.5)	22 (11.5)	26 (12.7)	8 (15.4)
Borrmann classification				
I	31 (6.9)	17 (8.9)	12 (5.9)	2 (3.8)
II	86 (19.2)	38 (19.8)	41 (20.0)	7 (13.5)
III	179 (39.9)	73 (38.0)	87 (42.4)	19 (36.5)
IV	98 (21.8)	45 (23.4)	40 (19.5)	13 (25.0)
Unknown	55 (12.2)	19 (9.9)	25 (12.2)	11 (21.2)
Lauren classification				
Intestinal type	28 (6.2)	12 (6.3)	13 (6.3)	3 (5.8)
Diffuse type	78 (17.4)	30 (15.6)	40 (19.5)	8 (15.4)
Mixed type	39 (8.7)	25 (13.0)	11 (5.4)	3 (5.8)
Unknown	304 (67.7)	125 (65.1)	141 (68.8)	38 (73.1)
pT				
T1	21 (4.7)	8 (4.2)	11 (5.4)	2 (3.8)
T2	19 (4.2)	11 (5.7)	6 (2.9)	2 (3.8)
T3	64 (14.3)	32 (16.7)	28 (13.7)	4 (7.7)
T4	339 (75.5)	138 (71.9)	158 (77.1)	43 (82.7)
Unknown	6 (1.3)	3 (1.6)	2 (1.0)	1 (1.9)
Number of nodes retrieved (mean ± SD)	23.3 ± 14.1	24.4 ± 14.3	23.1 ± 13.7	20.0 ± 15.0
Number of nodes metastasis (mean ± SD)	11.5 ± 11.0	12.1 ± 11.7	11.5 ± 10.8	8.6 ± 8.4
pN				
N0	55 (12.2)	21 (10.9)	27 (13.2)	7 (13.5)
N1	55 (12.2)	27 (14.1)	22 (10.7)	6 (11.5)
N2	77 (17.1)	29 (15.1)	39 (19.0)	9 (17.3)
N3	255 (56.8)	113 (58.9)	114 (55.6)	28 (53.8)
Unknown	7 (1.6)	2 (1.0)	3 (1.5)	2 (3.8)
pTNM				
I	23 (5.1)	8 (4.2)	11 (5.4)	4 (7.7)
II	41 (9.1)	24 (12.5)	14 (6.8)	3 (5.8)
III	315 (70.2)	132 (68.8)	149 (72.7)	34 (65.4)
IV	42 (9.4)	15 (7.8)	18 (8.8)	9 (17.3)
Unknown	28 (6.2)	13 (6.8)	13 (6.3)	2 (3.8)
Lymphatic vessel invasion				
Negative	184 (41.0)	90 (46.9)	75 (36.6)	19 (36.5)
Positive	217 (48.2)	86 (44.8)	109 (53.2)	22 (42.3)
Unknown	48 (10.7)	16 (8.3)	21 (10.2)	11 (21.2)
Blood vessel invasion				
Negative	185 (41.2)	90 (46.9)	76 (37.1)	19 (36.5)
Positive	216 (48.1)	86 (44.8)	108 (52.7)	22 (42.3)
Unknown	48 (10.7)	16 (8.3)	21 (10.2)	11 (21.2)
Nerve invasion				
Negative	292 (65.0)	126 (65.6)	138 (67.3)	28 (53.8)
Positive	113 (25.2)	52 (27.1)	47 (22.9)	14 (26.9)
Unknown	44 (9.8)	14 (7.3)	20 (9.8)	10 (19.2)
Extent of resection				
R1	412 (91.8)	180 (93.8)	188 (91.7)	44 (84.6)
R2	37 (8.2)	12 (6.2)	17 (8.3)	8 (15.4)
Combined resection				
No	337 (75.1)	143 (74.5)	152 (74.1)	42 (80.8)
Yes	112 (24.9)	49 (25.5)	53 (25.9)	10 (19.2)
Postoperative complications				
No	336 (74.8)	145 (75.5)	158 (77.1)	33 (63.5)
Yes	59 (13.1)	27 (14.1)	25 (12.2)	7 (13.5)
Unknown	54 (12.0)	20 (10.4)	22 (10.7)	12 (23.1)
Postoperative treatment				
No	52 (11.6)	23 (12.0)	23 (11.2)	6 (11.5)
Chemotherapy	158 (35.2)	71 (37.0)	72 (35.1)	15 (28.8)
Chemoradiotherapy	35 (7.8)	15 (7.8)	16 (7.8)	4 (7.7)
Reoperation	15 (3.3)	6 (3.1)	6 (3.0)	3 (5.8)
Unknown	189 (42.1)	77 (40.1)	88 (42.9)	24 (46.2)

*PG, proximal gastrectomy; DG, distal gastrectomy; TG, total gastrectomy; BMI, body mass index.

### Safety and Complications of Surgery

Open and laparoscopic approaches were used in 370 (82.4%) and 79 (17.6%) patients, respectively. One hundred thirty-five patients (23.4%) required intraoperative blood transfusion (**Supplementary Table 2**). The postoperative total complication rate was 13.1%. The overall mean duration of hospital stay was 15.9 days. Major postoperative complications are defined as anastomotic leak, hemorrhage, infection, gastroparesis, and intestinal obstruction. The specific information is shown in **Supplementary Table 3**.

### Survival Analysis of Prognostic Factors for Overall Survival and Recurrence-Free Survival

**Figure 1:**
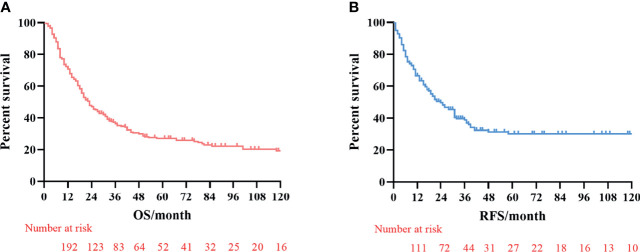
Kaplan–Meier survival curves of overall survival (OS) and recurrence-free survival (RFS). **(A)** Analysis of overall survival curve. **(B)** Analysis of the recurrence-free survival curve.

**Figure 2:**
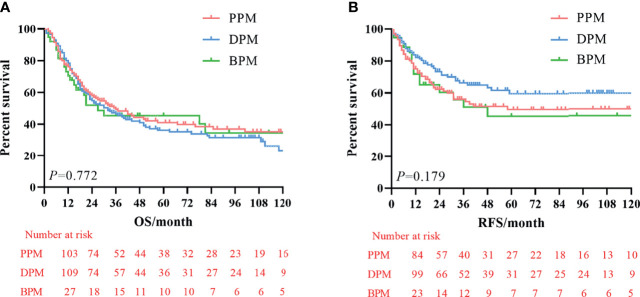
Kaplan–Meier survival curve of overall survival (OS) and recurrence-free survival (RFS) among three groups. **(A)** Analysis of overall survival curve. **(B)** Analysis of the recurrence-free survival curve.

The median survival time was 23.3 months (range 1–229 months). The 3- and 5-year OS rates for the total patients investigated were 47.5% and 39.3%, respectively, and the 3- and 5-year RFS rates were 60.0% and 53.6%, respectively. The survival curve is depicted in **Figure 1**. There was no significant difference in the survival curves among the PPM group, DPM group, and BPM group (**Figure 2**). Multivariate Cox regression analysis proved total gastrectomy (HR: 1.783, 95%CI: 1.133–2.805, *p* = 0.012), pT4 (HR: 5.264, 95%CI: 1.493–18.565, *p* = 0.01), pN2 (HR: 2.263, 95%CI: 1.164–4.397, *p* = 0.016), pN3 (HR: 2.327, 95%CI: 1.233–4.393, *p* = 0.009), and combined resection (HR: 1.952, 95%CI: 1.256–3.034, *p* = 0.003) to be independent risk factors of OS, while adjuvant treatment (HR: 0.540, 95%CI: 0.328–0.888, *p* = 0.015) to be an independent protective factor (**Table 2**). Meanwhile, multivariate analysis indicated pT3 (HR: 9.257, 95%CI: 1.152–74.386, *p* = 0.036) and pT4 (HR: 11.361, 95%CI: 1.469–87.847, *p* = 0.020) were independent risk factors for RFS, while age (HR: 0.979, 95%CI: 0.962–0.997, *p* = 0.023) and postoperative chemotherapy (HR: 0.315, 95%CI: 0.189–0.537, *p* < 0.001) were associated with improved RFS (**Table 3**).

**Table 2:** Univariate and multivariate Cox regression analyses of the predictors of OS in total patients.

Characteristic	Univariate analysis		Multivariate analysis	
	HR [95%CI]	*P*	HR [95%CI]	*P*
Age (mean±SD)	0.995[0.983-1.008]	0.436		
BMI (mean±SD)	1.016[0.977-1.057]	0.425		
Gender				
Male	Reference			
Female	1.136[0.830-1.556]	0.426		
Tumor location				
Proximal	Reference			
Distal	1.123[0.830-1.519]	0.451		
Middle	1.306[0.721-2.365]	0.379		
Unknown	2.182[0.302-15.766]	0.439		
Neoadjuvant therapy				
No	Reference			
Yes	1.313[0.846-2.038]	0.224		
Unknown	0.879[0.499-1.549]	0.656		
Gastric stump carcinoma				
No	Reference			
Yes	0.861[0.453-1.633]	0.646		
Unknown	0.533[0.370-0.770]	0.001		
Resection type				
PG	Reference		Reference	
DG	0.945[0.669-1.335]	0.749	1.476[0.967-2.251]	0.071
TG	1.600[1.098-2.332]	0.014	1.783[1.133-2.805]	0.012
Unknown	0.862[0.513-1.448]	0.574	0.870[0.394-1.925]	0.732
Surgical approach				
Open	Reference			
Laproscope	1.067[0.757-1.504]	0.710		
Tumor size (pathology)				
<5cm	Reference		Reference	
≥5cm	0.667[0.415-1.072]	0.094	0.908[0.597-1.382]	0.653
Unknown	1.056[0.716-1.558]	0.784	1.877[1.000-3.526]	0.050
Differentiation				
Well and Moderate	Reference		Reference	
Poor and Undifferentiated	2.124[1.284-3.515]	0.003	1.308[0.748-2.290]	0.346
Unknown	1.761[0.953-3.254]	0.071	1.548[0.737-3.252]	0.248
Borrman classification				
I	Reference			
II	0.700[0.366-1.339]	0.281		
III	1.131[0.630-2.031]	0.679		
IV	1.200[0.648-2.222]	0.563		
Unknown	0.483[0.240-0.972]	0.042		
Lauren classification				
intestinal type	Reference		Reference	
diffuse type	2.321[1.207-4.461]	0.012	1.504[0.737-3.072]	0.262
mixed type	1.653[0.774-3.531]	0.194	1.100[0.490-2.466]	0.818
Unknown	1.746[0.938-3.247]	0.079	1.189[0.611-2.312]	0.610
pT stage				
T1	Reference		Reference	
T2	1.520[0.340-6.793]	0.584	1.517[0.311-7.386]	0.606
T3	4.325[1.321-14.153]	0.015	3.283[0.893-12.078]	0.074
T4	7.945[2.532-24.934	<0.001	5.264[1.493-18.565]	0.010
Unknown	1.571[0.163-15.103]	0.696	1.109[0.098-12.549]	0.933
Number of nodes retrived (mean±SD)	0.996[0.985-1.006]	0.432		
pN stage				
N0	Reference		Reference	
N1	1.745[0.904-3.368]	0.097	1.312[0.640-2.689]	0.459
N2	2.833[1.566-5.124]	0.001	2.263[1.164-4.397]	0.016
N3	3.702[2.191-6.256]	<0.001	2.327[1.233-4.393]	0.009
Unknown	4.877[1.627-14.621]	0.005	2.812[0.733-10.781]	0.132
Lymphatic vessels invasion				
Negative	Reference		Reference	
Positive	1.505[1.108-2.042]	0.009	0.997[0.700-1.421]	0.988
Unknown	0.971[0.601-1.568	0.903	0.547[0.267-1.122]	0.100
Nerve invasion				
Negative	Reference			
Positive	1.222[0.891-1.675]	0.213		
Unknown	0.870[0.545-1.388]	0.558		
Margin involved				
Proximal	Reference			
Distal	1.115[0.827-1.505]	0.475		
Both	1.039[0.642-1.679]	0.877		
Combined resection				
No	Reference		Reference	
Yes	1.571[1.115-2.215]	0.010	1.952[1.256-3.034]	0.003
Postoperative complications				
No	Reference			
Yes	1.131[0.722-1.771]	0.591		
Unknown	0.918[0.601-1.404]	0.694		
Postoperative treatment				
No	Reference		Reference	
Yes	0.832[0.541-1.279]	0.402	0.540[0.328-0.888]	0.015
Chemotherapy	0.805[0.518-1.252]	0.336	0.655[0.403-1.066]	0.089
Chemoraidotherapy	1.006[0.576-1.757]	0.982	0.731[0.389-1.305]	0.273
Reoperation	1.132[0.492-2.603]	0.770	1.337[0.486-3.678]	0.574
Unknown	1.215[0.654-2.258]	0.825	0.847[0.517-1.389]	0.511

PG, proximal gastrectomy; DG, distal gastrectomy; TG, total gastrectomy; OS, overall survival; BMI, body mass index; HR, hazard ratio.

**Table 3:** Univariate and multivariate Cox regression analyses of the predictors of RFS in total patients.

Characteristic	Univariate analysis		Multivariate analysis	
	HR [95%CI]	*p*	HR [95%CI]	*p*
Age (mean ± SD)	0.986 [0.970–1.001]	0.070	0.979 [0.962–0.997]	0.023
BMI (mean ± SD)	0.977 [0.926–1.031]	0.397		
Gender				
Male	Reference			
Female	0.770 [0.490–1.209]	0.256		
Tumor location				
Proximal	Reference			
Distal	0.916 [0.624–1.345]	0.656		
Middle	0.653 [0.235–1.818]	0.414		
Unknown	48.848 [5.811–410.604]	<0.001		
Neoadjuvant therapy				
No	Reference			
Yes	1.428 [0.810–2.516]	0.218		
Unknown	2.072 [1.195–3.593]	0.009		
Gastric stump carcinoma				
No	Reference		Reference	
Yes	1.962 [1.071–3.593]	0.029	0.997 [0.423–2.352]	0.995
Unknown	0.493 [0.292–0.831]	0.008	0.404 [0.229–0.712]	0.002
Resection type				
PG	Reference			
DG	0.874 [0.554–1.381]	0.565		
TG	1.098 [0.620–1.942]	0.749		
Unknown	1.911 [1.133–3.224]	0.015		
Surgical approach				
Open	Reference		Reference	
Laproscope	1.557 [1.033–2.348]	0.035	1.145 [0.664–1.973]	0.626
Tumor size (pathology)				
<5 cm	Reference		Reference	
≥5 cm	2.288 [1.312–3.990]	0.004	1.327 [0.707–2.491]	0.378
Unknown	2.975 [1.567–5.650]	0.001	1.567 [0.661–3.712]	0.308
Differentiation				
Well and moderate	Reference			
Poor and undifferentiated	1.504 [0.817–2.768]	0.190		
Unknown	2.307 [1.151–4.62]	0.018		
Borrmann classification				
I	Reference			
II	1.106 [0.480–2.552]	0.813		
III	0.732 [0.322–1.667]	0.458		
IV	1.360 [0.592–3.123]	0.469		
Unknown	1.114 [0.477–2.600]	0.803		
Lauren classification				
Intestinal type	Reference			
Diffuse type	1.314 [0.580–2.976]	0.512		
Mixed type	1.166 [0.437–3.109]	0.759		
Unknown	1.580 [0.762–3.277]	0.219		
pT				
T1	Reference		Reference	
T2	5.125 [0.573–45.859]	0.144	3.347 [0.362–30.977]	0.287
T3	7.179 [0.947–54.395]	0.056	9.257 [1.152–74.386]	0.036
T4	14.103 [1.962–101.386]	0.009	11.361 [1.469–87.847]	0.020
Unknown	26.589 [2.758–256.350]	0.005	10.598 [0.998–112.496]	0.050
Number of nodes retrieved (mean ± SD)	0.993 [0.979–1.008]	0.348		
pN				
N0	Reference		Reference	
N1	1.954 [0.971–3.933]	0.060	1.791 [0.837–3.833]	0.133
N2	1.503 [0.740–3.052]	0.260	1.259 [0.566–2.800]	0.571
N3	2.115 [1.178–3.797]	0.012	1.570 [0.766–3.219]	0.218
Unknown	49.193 [14.481–167.111]	<0.001	16.404 [4.306–62.491]	<0.001
Lymphatic vessel invasion				
Negative	Reference		Reference	
Positive	1.523 [1.001–2.316]	0.049	1.215 [0.742–1.988]	0.439
Unknown	1.937 [1.142–3.286]	0.014	1.448 [0.691–3.034]	0.327
Nerve invasion				
Negative	Reference			
Positive	1.142 [0.740–1.762]	0.549		
Unknown	1.717 [1.046–2.821]	0.033		
Margin involved				
Proximal	Reference		Reference	
Distal	0.711 [0.478–1.059]	0.094	0.630 [0.414–0.960]	0.032
Both	1.056 [0.596–1.872]	0.851	0.767 [0.410–1.437]	0.408
Combined resection				
No	Reference			
Yes	1.381 [0.871–2.189]	0.170		
Postoperative complications				
No	Reference			
Yes	1.006 [0.536–1.889]	0.985		
Unknown	1.475 [0.913–2.385]	0.113		
Postoperative treatment				
No	Reference		Reference	
Yes	0.442 [0.285–0.686]	<0.001	0.324 [0.192–0.546]	<0.001
Chemotherapy	0.399 [0.251–0.635]	<0.001	0.315 [0.189–0.537]	<0.001
Chemoradiotherapy	0.660 [0.363–1.197]	0.171	0.604 [0.311–1.170]	0.135
Reoperation	0.716 [0.278–1.849]	0.491	0.420 [0.128–1.383]	0.154
Unknown	0.205 [0.113–0.371]	<0.001	0.187 [0.099–0.352]	<0.001

PG, proximal gastrectomy; DG, distal gastrectomy; TG, total gastrectomy; RFS, recurrence-free survival; BMI, body mass index; HR, hazard ratio.

### Subgroup Survival Analysis According to the Location of Positive Margin

In the PPM group, multivariate analysis demonstrated that neoadjuvant therapy (HR: 2.395, 95%CI: 1.174–4.887, *p* = 0.016) and pN3 (HR: 3.471, 95%CI: 1.057–11.396, *p* = 0.040) were independent risk factors for OS, while postoperative chemotherapy (HR: 0.457, 95%CI: 0.224–0.935, *p* = 0.032) was a protective factor. Regarding the RFS, pN1 (HR: 3.396, 95%: 1.071–10.771, *p* = 0.038) and pN3 (HR: 3.303, 95%CI: 1.132–9.635, *p* = 0.029) were independent risk factors, while postoperative chemotherapy (HR: 0.215, 95%CI: 0.108–0.428, *p* < 0.001) was a protective factor (**Tables 4A**, **4B**).

**Table 4a:** Multivariate Cox regression analyses of the predictors of OS in PPM group.

Prognostic factors	Unadjusted		Adjusted	
	HR [95%CI]	*p*	HR [95%CI]	*p*
Neoadjuvant chemotherapy				
No	Reference		Reference	
Yes	1.875 [1.040–3.382]	0.037	2.395 [1.174–4.887]	0.016
pN				
N0	Reference		Reference	
N1	2.026 [0.623–6.590]	0.241	1.473 [0.411–5.275]	0.552
N2	4.032 [1.311–12.401]	0.015	3.232 [0.940–11.109]	0.063
N3	5.515 [1.989–15.286]	0.001	3.471 [1.057–11.396]	0.040
Postoperative treatment				
No	Reference		Reference	
Yes	0.509 [0.269–0.962]	0.038	0.466 [0.234–0.927]	0.030
Chemotherapy	0.511 [0.270–0.968]	0.039	0.457 [0.224–0.935]	0.032
Chemoradiotherapy	0.712 [0.298–1.701]	0.444	0.631 [0.245–1.625]	0.540
Reoperation	0.574 [0.130–2.532]	0.464	0.571 [0.080–4.065]	0.576
Unknown	0.801 [0.419–1.534]	0.504	0.946 [0.449–1.995]	0.885

HRs were adjusted for resection type, tumor size (pathology), differentiation, Lauren classification, pT stage, and lymphatic vessel invasion.

OS, overall survival; PPM, proximal positive margin; HR, hazard ratio.

**Table 4b:** Multivariate Cox regression analyses of the predictors of RFS in PPM group.

Prognostic factors	Unadjusted		Adjusted	
	HR [95%CI]	*p*	HR [95%CI]	*p*
pN				
N0	Reference		Reference	
N1	2.443 [0.848–7.041]	0.098	3.396 [1.071–10.771]	0.038
N2	1.548 [0.491–4.885]	0.456	1.559 [0.406–5.981]	0.518
N3	2.459 [0.948–6.376]	0.064	3.303 [1.132–9.635]	0.029
Postoperative treatment				
No	Reference		Reference	
Yes	0.296 [0.161–0.542]	<0.001	0.296 [0.138–0.524]	<0.001
Chemotherapy	0.262 [0.138–0.498]	<0.001	0.215 [0.108–0.428]	<0.001
Chemoradiotherapy	0.526 [0.226–1.222]	0.135	0.527 [0.198–1.399]	0.198
Reoperation	0.477 [0.139–1.635]	0.239	0.264 [0.047–1.482]	0.130
Unknown	0.071 [0.024–0.211]	<0.001	0.069 [0.022–0.214]	<0.001

HRs were adjusted for gastric stump carcinoma, surgical approach, and tumor size (pathology).

RFS, recurrence-free survival; PPM, proximal positive margin; HR, hazard ratio.

In the DPM group, multivariate analysis demonstrated that body mass index (BMI) (HR: 1.075, 95%CI: 1.005–1.151, *p* = 0.037) is an independent risk factor for OS. Regarding the RFS, lymphatic vessel invasion (HR: 2.733, 95%: 1.082–6.903, *p* = 0.033) was an independent risk factor, while age (HR: 0.959, 95%CI: 0.929–0.989, *p* = 0.008) and postoperative chemotherapy (HR: 0.365, 95%CI: 0.144–0.929, *p* = 0.035) were protective factors (**Tables 4C**, **4D**).

**Table 4c:** Multivariate Cox regression analyses of the predictors of OS in DPM group.

Prognostic factors	Unadjusted		Adjusted	
	HR [95%CI]	*p*	HR [95%CI]	*p*
BMI (mean ± SD)	1.059 [0.995–1.126]	0.070	1.075 [1.005–1.151]	0.037
Postoperative treatment				
No	Reference		Reference	
Yes	1.231 [0.623–2.429]	0.550	0.503 [0.191–1.324]	0.164
Chemotherapy	1.231 [0.613–2.472]	0.559	0.801 [0.327–1.960]	0.627
Chemoradiotherapy	1.284 [0.554–2.973]	0.560	0.811 [0.294–2.237]	0.685
Reoperation	4.454 [1.375–14.430]	0.013	4.800 [0.999–23.072]	0.050
Unknown	1.201 [0.581–2.483]	0.621	0.637 [0.256–1.583]	0.331

HRs were adjusted for age, Borrmann classification, pT stage, pN stage, lymphatic vessel invasion, and combined resection.

OS, overall survival; DPM, distal positive margin; HR, hazard ratio; BMI, body mass index.

**Table 4d:** Multivariate Cox regression analyses of the predictors of RFS in DPM group.

Prognostic factors	Unadjusted		Adjusted	
	HR [95%CI]	*p*	HR [95%CI]	*p*
Age (mean ± SD)	0.970 [0.947–0.993]	0.012	0.959 [0.929–0.989]	0.008
Lymphatic vessel invasion				
Negative	Reference		Reference	
Positive	1.925 [0.957–3.871]	0.066	2.733 [1.082–6.903]	0.033
Postoperative treatment				
No	Reference		Reference	
Yes	0.553 [0.270–1.133]	0.105	0.322 [0.132–0.785]	0.013
Chemotherapy	0.527 [0.248–1.119]	0.095	0.365 [0.144–0.929]	0.035
Chemoradiotherapy	0.628 [0.231–1.706]	0.361	0.594 [0.194–1.813]	0.360
Reoperation	1.494 [0.326–6.860]	0.605	0.613 [0.106–3.537]	0.585
Unknown	0.277 [0.107–0.714]	0.008	0.210 [0.070–0.628]	0.005

HRs were adjusted for pT stage and pN stage.

RFS, recurrence-free survival; DPM, distal positive margin; HR, hazard ratio.

In the BPM group, multivariate analysis demonstrated that pN3 (HR: 15.544, 95%CI: 1.354–178.394, *p* = 0.028) was an independent risk factor for OS. There were no prognostic factors identified for RFS (**Tables 4E**, **4F**).

**Table 4e:** Multivariate Cox regression analyses of the predictors of OS in BPM group.

Prognostic factors	Unadjusted		Adjusted	
	HR [95%CI]	*p*	HR [95%CI]	*p*
pN				
N0	Reference		Reference	
N1	6.625 [0.591–74.244]	0.125	10.657 [0.650–174.711]	0.097
N2	3.949 [0.441–35.373]	0.220	5.285 [0.449–62.238]	0.186
N3	8.690 [1.131–66.752]	0.038	15.544 [1.354–178.394]	0.028
Postoperative treatment				
No	Reference		Reference	
Yes	0.931 [0.256–3.395]	0.914	0.972 [0.252–3.749]	0.968
Chemotherapy	0.867 [0.224–3.364]	0.837	0.910 [0.228–3.617]	0.893
Chemoradiotherapy	1.122 [0.225–5.595]	0.888	1.608 [0.269–9.614]	0.602
Reoperation	0.465 [0.048–4.494]	0.508	5.710 [0.367–88.879]]	0.213
Unknown	0.799 [0.206–3.100]	0.745	0.941 [0.238–3.721]	0.931

OS, overall survival; BPM, bilateral positive margin; HR, hazard ratio.

**Table 4f:** Multivariate Cox regression analyses of the predictors of RFS in BPM group.

Prognostic factors	Unadjusted		Adjusted	
	HR [95%CI]	*p*	HR [95%CI]	*p*
Postoperative treatment				
No	Reference		Reference	
Yes	0.964 [0.199–4.662]	0.964	0.964 [0.199–4.662]	0.964
Chemotherapy	0.675 [0.123–3.705]	0.651	0.675 [0.123–3.705]	0.651
Chemoradiotherapy	2.234 [0.369–13.510]	0.381	2.234 [0.369–13.510]	0.381
Reoperation	NA	0.988	NA	0.988
Unknown	1.045 [0.210–5.207]	0.957	1.045 [0.210–5.207]	0.957

NA, not available; RFS, recurrence-free survival; BPM, bilateral positive margin; HR, hazard ratio.

### Recurrence Patterns

**Figure 3:**
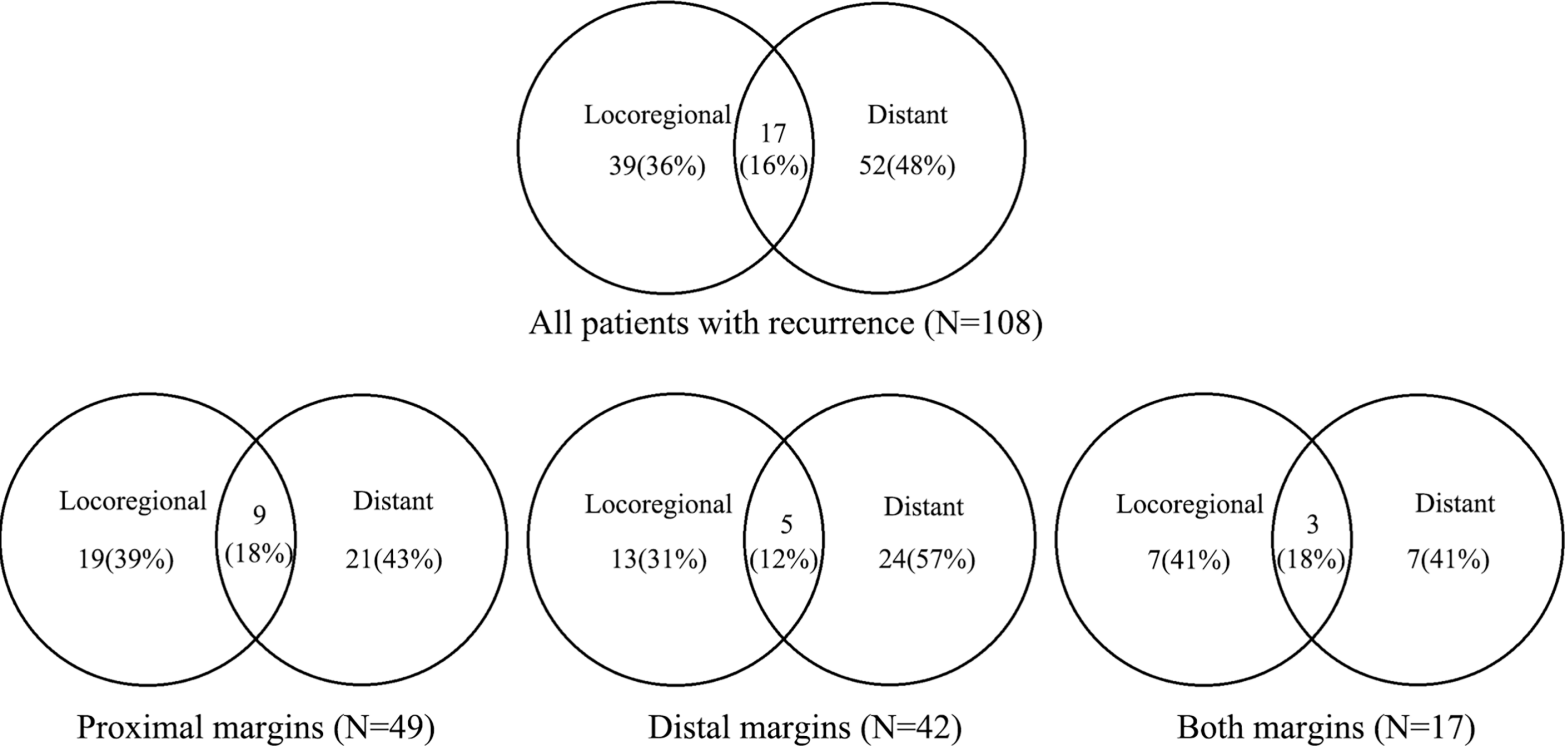
Proportion of first recurrence sites and recurrence patterns in patients with positive margin.

During follow-up, a total of 118 patients (26.3%) developed recurrence. Among 108 patients where the site of recurrence was known, 39 patients (36.1%) had locoregional recurrence, 52 patients (48.1%) had a distant recurrence, and 17 patients (15.7%) had mixed recurrence (**Figure 3**). The major locoregional recurrence sites were peritoneal (29.6%), locoregional lymph nodes (25.0%), and remnant stomach (21.3%). The main distant recurrence sites were the liver (23.1%) and supraclavicular lymph nodes (8.3%).

## Discussion

Several studies have reported that PM was an independent adverse prognostic factor compared with a negative margin in patients with GC, even though the incidence of PM was relatively low (5–7, 20–22). Considering the controversy of the adjuvant treatment options, we conducted this study. In the present study, we found that the advanced pathologic stage was associated with poor prognosis, while postoperative adjuvant chemotherapy might prolong the RFS in PM patients after gastrectomy, especially for patients with PPM and DPM. The current data might provide some clues for clinical practice in the future.

Several previous studies have revealed that postoperative adjuvant treatment may improve the survival of PM patients (11, 14, 16). On the contrary, a recent retrospective study of 69 PM patients with gastric and esophageal adenocarcinoma found that adjuvant treatment did not appear to confer RFS or OS benefits (*p* = 0.26 and *p* = 0.83, respectively) (18). In our study, we found that postoperative therapy was strongly associated with increased RFS and OS. Previous findings have shown that the impact of PM on prognosis was more pronounced in patients with lower stages (6, 9, 10, 22, 23). Liang and his team also reported that the OS of patients with PM was similar to that of the patients staged IIIc with R0 resection (6). Thus, PM patients after gastrectomy might be considered as stage IIIc and stage IV to some extent. Given this, postoperative adjuvant treatment may be helpful in improving RFS and OS in PM patients. However, the different choices of adjuvant treatments and a limited number of PM patients may be responsible for the survival discrepancies among different studies.

In the NCCN guideline, adjuvant CCRT is recommended for PM patients (12). The investigators of Leiden University Medical Center reported the results of adjuvant CCRT in the treatment of 22 patients with R1 resection, and the results demonstrated that the 2-year OS was significantly higher in the adjuvant CCRT group compared with the surgery-only group (66% vs. 29%; HR: 2.91; *p* = 0.002) (16). Similarly, in an NCDB analysis conducted in 2016 in 1,021 PM patients who were treated with adjuvant CCRT (501 patients) or adjuvant chemotherapy (520 patients), the investigators reported that adjuvant CCRT was associated with higher OS (HR: 0.72; 95%CI: 0.58–0.91; *p* = 0.005) (14). In addition, Zhang and his team found that the 3-year RFS rate and OS were higher in the adjuvant CCRT group (33 patients) compared with the adjuvant chemotherapy group (81 patients) in PM patients (45.1% vs. 38.1%, *p* = 0.09; 49.6% vs. 39.4%, *p* = 0.20, respectively) (11). However, according to Ma et al., adjuvant CCRT did not induce a statistically significant difference in OS (18). The results of the current study also revealed that adjuvant CCRT did not improve any survival outcome in PM patients.

In the present study, we found that adjuvant chemotherapy was associated with better RFS. Although the postoperative chemotherapy was not significantly correlated with OS, the trend of better OS was still observed. When we performed subgroup analysis according to the location of PM, we found that postoperative chemotherapy could significantly prolong RFS in both the PPM group and BPM group while prolonging OS in the PPM group. However, adjuvant therapy showed no survival benefit in the BPM group. At present, few studies indicated that adjuvant chemotherapy alone could prolong survival in PM patients after gastrectomy. Speculatively, differences in study populations may account for the discrepancy in results. Firstly, the proportion of the Asian population with PM in the NCDB database is only 6.7% (68/1021) (14), while the patients in our study are Chinese. Secondly, in the study of Ma et al., 11 (16%) cases of esophageal cancer and 28 (41%) cases of esophageal gastric junction cancer patients were included (18). Thirdly, in the study of Zhou et al., PM patients were further restricted; and patients with remnant GC, patients who received neoadjuvant therapy, and patients with positive peritoneal lavage cytology were excluded (11). Fourthly, in the BPM group, the proportion of stage IV patients was higher than that of other groups. It might be a reason that postoperative chemotherapy was not identified as the protective factor for OS and RFS.

The NCCN guideline indicated that “reoperation, if feasible, can also be considered following R1 resection” (12). In contrast, our study found that PM patients who received reoperation did not increase survival time and achieved worse OS in the DPM group. Notably, evidence showed that re-excision for intraoperatively PM to negative margin improved the prognosis of the patients with advanced GC, especially in those patients with ≤pN2-category disease (median survival of 44 vs. 25 months; *p* = 0.021) (24). The reason for this is probably the secondary trauma caused by the reoperation, which may affect the timing of postoperative adjuvant treatment. Thus, intraoperative re-excision is necessary, while the reoperation should be carefully selected in PM patients. Due to the limited number of cases, more evidence is required to clarify this issue.

The rate of recurrence was previously reported to be significantly higher in PM patients (63.6%~76%) (5, 18, 20, 22). Furthermore, the recurrence patterns were not universally identical. Concretely, the site of the first recurrence was distant in 40.8%~72% of patients, locoregional in 14.3%~29.6% of patients, and mixed in 12%~29.6% of patients (5, 18, 20, 22). In our study, the overall recurrence rate was lower (26.3%), and the rate of locoregional recurrence was much higher (36.1%). The results were similar to the recurrence patterns of negative margin (31.4%~39.7%) (5, 8, 20, 22). Multiple potential reasons may be responsible for the discrepancies. Firstly, a previous study has demonstrated that compared with that of negative margin patients, the recurrence rate of PM patients was only significantly increased in pT1–2, pN0–1, and I–II stage patients, but not in pT3–4, pN2–3, and III–VI stage patients (22). However, the majority of patients enrolled in our study have advanced T or N stage. Secondly, the median follow-up time of 23.3 months is relatively short for PM patients, and the risk of recurrence may increase with longer follow-up.

In our study, we found that pT4, pN2, and pN3 were independent risk factors for OS in PM patients. In subgroup analysis, only pN3 was observed to be the main independent prognostic element for OS in the PPM group. It is perhaps worth noting that previous findings have shown that the impact of PM on prognosis was more pronounced in patients with a lower stage (6, 9, 20, 22, 23). On the other hand, Endo et al. (23) and Songun et al. (22) have found that survival might be equivalent between negative margin and PM in patients with positive peritoneal lavage cytology (CY1). This may suggest that among patients with PM in advanced GC, a competitive relationship might exist between positive surgical margin and tumor staging on survival. In addition, we have found that the prognostic factors were different among the PPM group, DPM group, and BPM group. In our previous study, we found that the clinicopathological characteristics of proximal GC (PGC) patients presented differently with distal GC (DGC) patients (19). Therefore, we speculated that the tumor biology was different between PGC and DGC. These discrepancies may be the major reason accounting for the difference of prognostic factors among the PPM group, DPM group, and BPM group.

The previous prospective clinical trials have demonstrated that neoadjuvant therapy could improve the survival of patients with locally advanced GC (25–28). In these studies, the patients with PM were not been excluded. However, in the present study, we found that neoadjuvant therapy was an independent factor in the OS of the PPM group. One possible reason for the discrepancy was that patients who received neoadjuvant treatment had a higher stage. Furthermore, significant consideration should be given to the Tumor Regression Grade (TRG) after neoadjuvant treatment. A previous study has demonstrated that patients with TRG4–5 had worse survival than TRG1–3 (29). Patients with PM might be likely to have higher TRG (TRG4–5). Therefore, neoadjuvant therapy might be an independent risk factor for the prognosis in PM patients. Further studies are needed to clarify this hypothesis.

The strengths and limitations should be assessed objectively. The present study was the largest sample study in a single center focusing on PM patients with GC. Our study, for the first time, reported that adjuvant chemotherapy could result in a survival benefit, while the postoperative adjuvant CCRT may not be necessary for PM patients. In addition, we performed subgroup analysis according to the location of the positive resection margin. However, there are certain limitations to our study. Firstly, this is a retrospective, single-center study, limiting the generalizability of the findings. Secondly, some patients had a relatively short follow-up period, which might lead to an underreported recurrence rate. Thirdly, many patients were followed up by other oncological centers; therefore, the adjuvant treatment strategies have not been well recorded. Fourthly, relatively few patients in the present study received adjuvant CCRT; and the specific chemotherapy regimen data, the radiotherapy dose, duration of chemotherapy, and data about adverse effects of chemotherapy might be important factors affecting results.

## Conclusion

In conclusion, postoperative adjuvant chemotherapy might prolong the RFS in PM patients after gastrectomy, especially for patients with PPM and DPM. Still, further prospective trials are warranted to verify and support our conclusions.

## Data Availability Statement

The raw data supporting the conclusions of this article will be made available by the authors, without undue reservation.

## Ethics Statement

The studies involving human participants were reviewed and approved by Institutional Review Board at the China National Cancer Center. Written informed consent for participation was not required for this study in accordance with the national legislation and the institutional requirements.

## Author Contributions

1) Guarantor of integrity of the entire study: YC and DZ. 2) Study concepts and design: XZ, YC, and DZ. 3) Provision of study materials or patients: XZ, LZ, and PN. 4) Collection and assembly of data: XZ, LZ, PN, TW, WW, CS, and ZL. 5) Statistical analysis: XZ, LZ, PN, and YC. 6) Manuscript preparation: All authors. 7) Manuscript editing: All authors.

## Conflict of Interest

The authors declare that the research was conducted in the absence of any commercial or financial relationships that could be construed as a potential conflict of interest.

## Publisher’s Note

All claims expressed in this article are solely those of the authors and do not necessarily represent those of their affiliated organizations, or those of the publisher, the editors and the reviewers. Any product that may be evaluated in this article, or claim that may be made by its manufacturer, is not guaranteed or endorsed by the publisher.
