# Supplementary material for: Adjuvant Chemotherapy Might Be Recommended to Patients With Positive Margin After Gastrectomy: A 20-Year Retrospective Analysis in a Single Center
Source: Front Oncol. 2022 Feb 2;11:794032. doi: 10.3389/fonc.2021.794032 (PMC8847385; doi:10.3389/fonc.2021.794032)
Supplement: Supplementary file 1 [file Table_1.docx]

**Appendix**

Supplementary Table 1: Detailed information of combined resection.

Supplementary Table 2: Detailed information of intraoperative conditions and postoperative recovery.

Supplementary Table 3: Detailed information of postoperative complications.

Supplementary Table 4: Univariate and multivariate Cox regression analyses of the predictors of OS in PPM group.

Supplementary Table 5: Univariate and multivariate Cox regression analyses of the predictors of RFS in PPM group.

Supplementary Table 6: Univariate and multivariate Cox regression analyses of the predictors of OS in DPM group.

Supplementary Table 7: Univariate and multivariate Cox regression analyses of the predictors of RFS in DPM group.

Supplementary Table 8: Univariate and multivariate Cox regression analyses of the predictors of OS in BPM group.

Supplementary Table 9: Univariate and multivariate Cox regression analyses of the predictors of RFS in BPM group.

Supplementary Table 1: Detailed information of combined resection.

| Combined Resection | | Total |
| --- | --- | --- |
|  |  | N=112(%) |
| Esophagus |  | 96(85.7) |
| Liver |  | 1(0.9) |
| Colon |  | 4(3.6) |
| Appendix |  | 1(0.9) |
| Accessories | | 1(0.9) |
| Lung |  | 1(0.9) |
| Gallbladder | | 1(0.9) |
| Spleen |  | 8(7.1) |
| Pancreas |  | 1(0.9) |

Supplementary Table 2: Detailed information of intraoperative conditions and postoperative recovery.

| Variables |  |  |  | Total | PPM | DPM | BPM |
| --- | --- | --- | --- | --- | --- | --- | --- |
|  |  |  |  | N=449(%) | N=192(%) | N=205(%) | N=52(%) |
| Mean operative time /min | | |  | 202.9 | 205.9 | 201.4 | 197.7 |
| Mean intraoperative blood loss /mL | | | | 286.2 | 323.5 | 259.5 | 245 |
| Blood transfusion | |  |  |  |  |  |  |
| No |  |  |  | 267(53.5) | 111(57.8) | 126(61.5) | 30(57.7) |
| Yes |  |  |  | 105(23.4) | 50(26.0) | 44(21.5) | 11(21.2) |
| Unknown |  |  |  | 77(17.1) | 31(16.1) | 35(17.1) | 11(21.2) |
| Blood transfusion /ml | |  |  | 818.4 | 850 | 784.6 | 833.3 |
| Mean postoperative hospital stay /d | | | | 15.9 | 15.6 | 15.5 | 18.7 |

Supplementary Table 3: Detailed information of postoperative complications.

| Postoperative complications | | | | | |  | Total | PPM | | | DPM | BPM | |  |
| --- | --- | --- | --- | --- | --- | --- | --- | --- | --- | --- | --- | --- | --- | --- |
|  |  | |  | | |  | N=449(%) | N=192(%) | | | N=205(%) | N=52(%) | |  |
| Anastomotic leak | | |  | | |  | 13(2.9) | 7(3.6) | | | 3(1.5) | 3(5.8) | |  |
| Intra-abdominal infections | | | | | |  | 20(4.5) | 9(4.7) | | | 8(3.9) | 3(5.8) | |  |
| Intra-abdominal hemorrhage | | | | | |  | 2(0.4) | 0(0.0) | | | 2(1.0) | 0(0.0) | |  |
| Gastrointestinal hemorrhage | | | | | |  | 7(1.6) | 4(2.1) | | | 3(1.5) | 0(0.0) | |  |
| Postoperative intestinal obstruction | | | | | | | 2(0.4) | 2(1.0) | | | 0(0.0) | 0(0.0) | |  |
| Gastroparesis | | |  | | |  | 7(1.6) | 3(1.6) | | | 3(1.5) | 1(1.9) | |  |
| Pulmonary complications | | | | | |  | 24(5.3) | 11(5.7) | | | 11(5.4) | 2(3.8) | |  |
| Fat liquefaction | | |  | | |  | 5(1.1) | 2(1.0) | | | 2(1.0) | 1(1.9) | |  |
| Other | |  | |  |  | | 2(0.4) | | 1(0.5) | 1(0.5) | | | 0(0.0) | |

Supplementary Table 4: Univariate and multivariate Cox regression analyses of the predictors of OS in PPM group.

| Characteristic | Univariate analysis | | Multivariate analysis | |
| --- | --- | --- | --- | --- |
|  | HR [95%CI] | *P* | HR [95%CI] | *P* |
| Age (mean±SD） | 1.003[0.983-1.024] | 0.746 |  |  |
| BMI (mean±SD） | 0.999[0.943-1.057] | 0.961 |  |  |
| Gender |  |  |  |  |
| Male | Reference |  |  |  |
| Female | 1.172[0.726-1.891] | 0.516 |  |  |
| Tumor location |  |  |  |  |
| Proximal | Reference |  |  |  |
| Distal | 1.299[0.822-2.052] | 0.262 |  |  |
| Middle | 1.264[0.529-3.022] | 0.598 |  |  |
| Unknown | NA |  |  |  |
| Neoadjuvant therapy |  |  |  |  |
| No | Reference |  | Reference |  |
| Yes | 1.875[1.040-3.382] | 0.037 | 2.395[1.174-4.887] | 0.016 |
| Unknown | 0.694[0.252-1.910] | 0.479 | 0.383[0.024-5.994] | 0.494 |
| Gastric stump carcinoma |  |  |  |  |
| No | Reference |  |  |  |
| Yes | 1.225[0.490-3.060] | 0.665 |  |  |
| Unknown | 0.766[0.445-1.319] | 0.337 |  |  |
| Resection type |  |  |  |  |
| PG | Reference |  | Reference |  |
| DG | 1.039[0.574-1.881] | 0.899 | 0.932[0.476-1.824] | 0.837 |
| TG | 2.082[1.228-3.530] | 0.006 | 1.559[0.843-2.883] | 0.157 |
| Unknown | 0.856[0.373-1.965] | 0.714 | 0.878[0.245-3.152] | 0.842 |
| Surgical approach |  |  |  |  |
| Open | Reference |  |  |  |
| Laproscope | 1.035[0.596-1.797] | 0.902 |  |  |
| Tumor size (pathology) |  |  |  |  |
| <5cm | Reference |  | Reference |  |
| ≥5cm | 1.829[1.034-3.234] | 0.038 | 0.962[0.471-1.963] | 0.915 |
| Unknown | 1.405[0.613-3.222] | 0.422 | 1.855[0.540-6.377] | 0.326 |
| Differentiation |  |  |  |  |
| Well and Moderate | Reference |  | Reference |  |
| Poor and Undifferentiated | 2.078[0.995-4.338] | 0.051 | 1.329[0.529-3.342] | 0.545 |
| Unknown | 1.558[0.582-4.175] | 0.378 | 2.442[0.525-11.357] | 0.255 |
| Borrman classification |  |  |  |  |
| Ⅰ | Reference |  |  |  |
| Ⅱ | 0.956[0.340-2.691] | 0.933 |  |  |
| Ⅲ | 1.408[0.549-3.612] | 0.477 |  |  |
| Ⅳ | 1.626[0.619-4.271] | 0.324 |  |  |
| Unknown | 0.691[0.210-2.271] | 0.543 |  |  |
| Lauren classification |  |  |  |  |
| intestinal type | Reference |  | Reference |  |
| diffuse type | 2.724[0.920-8.063] | 0.070 | 1.766[0.462-6.754] | 0.406 |
| mixed type | 1.716[0.545-5.405] | 0.356 | 0.914[0.225-3.710] | 0.900 |
| Unknown | 1.832[0.657-5.108] | 0.247 | 1.352[0.359-5.096] | 0.656 |
| pT stage |  |  |  |  |
| T1 | Reference |  | Reference |  |
| T2 | 2.773[0.310-24.838] | 0.362 | 2.447[0.223-26.852] | 0.464 |
| T3 | 3.209[0.417-24.693] | 0.263 | 2.208[0.247-19.731] | 0.478 |
| T4 | 7.698[1.065-55.621] | 0.043 | 4.624[0.533-40.154] | 0.165 |
| Unknown | NA | 0.974 | NA | 0.976 |
| Number of nodes retrived (mean±SD) | 1.005[0.990-1.020] | 0.502 |  |  |
| pN stage |  |  |  |  |
| N0 | Reference |  | Reference |  |
| N1 | 2.026[0.623-6.590] | 0.241 | 1.473[0.411-5.275] | 0.552 |
| N2 | 4.032[1.311-12.401] | 0.015 | 3.232[0.940-11.109] | 0.063 |
| N3 | 5.515[1.989-15.286] | 0.001 | 3.471[1.057-11.396] | 0.040 |
| Unknown | 105.110[16.335-676.199] | <0.001 | 78.689[8.869-698.173] | <0.001 |
| Lymphatic vessels invasion |  |  |  |  |
| Negative | Reference |  |  |  |
| Positive | 1.461[0.929-2.299] | 0.100 | 1.126[0.649-1.956] | 0.673 |
| Unknown | 0.702[0.274-1.796] | 0.460 | 1.133[0.116-11.083] | 0.915 |
| Nerve invasion |  |  |  |  |
| Negative | Reference |  |  |  |
| Positive | 1.051[0.653-1.692] | 0.838 |  |  |
| Unknown | 0.642[0.255-1.616] | 0.347 |  |  |
| Combined resection |  |  |  |  |
| No | Reference |  |  |  |
| Yes | 1.441[0.859-2.415] | 0.166 |  |  |
| Postoperative complications |  |  |  |  |
| No | Reference |  |  |  |
| Yes | 1.142[0.548-2.382] | 0.723 |  |  |
| Unknown | 0.634[0.275-1.463] | 0.285 |  |  |
| Postoperative treatment |  |  |  |  |
| No | Reference |  |  |  |
| Yes | 0.509[0.269-0.962] | 0.038 | 0.466[0.234-0.927] | 0.030 |
| Chemotherapy | 0.511[0.270-0.968] | 0.039 | 0.457[0.224-0.935] | 0.032 |
| Chemoraidotherapy | 0.712[0.298-1.701] | 0.444 | 0.631[0.245-1.625] | 0.540 |
| Reoperation | 0.574[0.130-2.532] | 0.464 | 0.571[0.080-4.065] | 0.576 |
| Unknown | 0.801[0.419-1.534] | 0.504 | 0.946[0.449-1.995] | 0.885 |

NA: Not Available;

Supplementary Table 5: Univariate and multivariate Cox regression analyses of the predictors of RFS in PPM group.

| Characteristic | Univariate analysis | | Multivariate analysis | |
| --- | --- | --- | --- | --- |
|  | HR [95%CI] | *P* | HR [95%CI] | *P* |
| Age (mean±SD） | 0.998[0.974-1.022] | 0.874 |  |  |
| BMI (mean±SD） | 1.003[0.935-1.077] | 0.923 |  |  |
| Gender |  |  |  |  |
| Male | Reference |  |  |  |
| Female | 0.683[0.352-1.324] | 0.259 |  |  |
| Tumor location |  |  |  |  |
| Proximal | Reference |  |  |  |
| Distal | 0.830[0.484-1.425] | 0.500 |  |  |
| Middle | 0.315[0.043-2.319 | 0.257 |  |  |
| Unknown | NA |  |  |  |
| Neoadjuvant therapy |  |  |  |  |
| No | Reference |  |  |  |
| Yes | 1.505[0.701-3.233] | 0.295 |  |  |
| Unknown | 1.759[0.783-3.950] | 0.171 |  |  |
| Gastric stump carcinoma |  |  |  |  |
| No | Reference |  |  |  |
| Yes | 2.242[1.049-4.791] | 0.037 | 1.409[0.410-4.845] | 0.587 |
| Unknown | 0.372[0.147-0.942] | 0.037 | 0.380[0.145-0.996] | 0.049 |
| Resection type |  |  |  |  |
| PG | Reference |  |  |  |
| DG | 0.718[0.348-1.482] | 0.371 |  |  |
| TG | 0.808[0.372-1.758] | 0.591 |  |  |
| Unknown | 1.610[0.795-3.259] | 0.186 |  |  |
| Surgical approach |  |  |  |  |
| Open | Reference |  |  |  |
| Laproscope | 1.988[1.114-3.547] | 0.020 | 1.833[0.907-3.909] | 0.089 |
| Tumor size (pathology) |  |  |  |  |
| <5cm | Reference |  | Reference |  |
| ≥5cm | 2.729[1.215-6.126] | 0.015 | 2.243[0.937-5.366] | 0.070 |
| Unknown | 3.230[1.224-8.527] | 0.018 | 1.416[0.450-4.455] | 0.552 |
| Differentiation |  |  |  |  |
| Well and Moderate | Reference |  |  |  |
| Poor and Undifferentiated | 1.375[0.612-3.091] | 0.440 |  |  |
| Unknown | 2.047[0.775-5.407] | 0.148 |  |  |
| Borrman classification |  |  |  |  |
| Ⅰ | Reference |  |  |  |
| Ⅱ | 1.165[0.429-3.158] | 0.765 |  |  |
| Ⅲ | 0.480[0.169-1.365] | 0.169 |  |  |
| Ⅳ | 0.600[0.201-1.793] | 0.360 |  |  |
| Unknown | 1.233[0.428-3.553] | 0.698 |  |  |
| Lauren classification |  |  |  |  |
| intestinal type | Reference |  |  |  |
| diffuse type | 2.260[0.477-10.708] | 0.304 |  |  |
| mixed type | 2.929[0.621-13.816] | 0.175 |  |  |
| Unknown | 2.705[0.650-11.253] | 0.171 |  |  |
| pT stage |  |  |  |  |
| T1 | Reference |  |  |  |
| T2 | NA | 0.905 |  |  |
| T3 | NA | 0.907 |  |  |
| T4 | NA | 0.896 |  |  |
| Unknown | NA | 0.897 |  |  |
| Number of nodes retrived (mean±SD) | 0.997[0.977-1.018] | 0.775 |  |  |
| pN stage |  |  |  |  |
| N0 | Reference |  | Reference |  |
| N1 | 2.443[0.848-7.041] | 0.098 | 3.396[1.071-10.771] | 0.038 |
| N2 | 1.548[0.491-4.885] | 0.456 | 1.559[0.406-5.981] | 0.518 |
| N3 | 2.459[0.948-6.376] | 0.064 | 3.303[1.132-9.635] | 0.029 |
| Unknown | 33.601[5.924-190.581] | <0.001 | 24.435[3.672-162.597] | 0.001 |
| Lymphatic vessels invasion |  |  |  |  |
| Negative | Reference |  |  |  |
| Positive | 1.300[0.724-2.333] | 0.380 |  |  |
| Unknown | 1.881[0.863-4.102] | 0.112 |  |  |
| Nerve invasion |  |  |  |  |
| Negative | Reference |  |  |  |
| Positive | 1.003[0.539-1.867] | 0.991 |  |  |
| Unknown | 1.840[0.870-3.891] | 0.110 |  |  |
| Combined resection |  |  |  |  |
| No | Reference |  |  |  |
| Yes | 1.431[0.764-2.680] | 0.263 |  |  |
| Postoperative complications |  |  |  |  |
| No | Reference |  |  |  |
| Yes | 0.691[0.214-2.235] | 0.537 |  |  |
| Unknown | 1.708[0.853-3.420 | 0.131 |  |  |
| Postoperative treatment |  |  |  |  |
| No | Reference |  |  |  |
| Yes | 0.296[0.161-0.542] | <0.001 | 0.296[0.138-0.524] | <0.001 |
| Chemotherapy | 0.262[0.138-0.498] | <0.001 | 0.215[0.108-0.428] | <0.001 |
| Chemoraidotherapy | 0.526[0.226-1.222] | 0.135 | 0.527[0.198-1.399] | 0.198 |
| Reoperation | 0.477[0.139-1.635] | 0.239 | 0.264[0.047-1.482] | 0.130 |
| Unknown | 0.071[0.024-0.211] | <0.001 | 0.069[0.022-0.214] | <0.001 |

NA: Not Available;

Supplementary Table 6 Univariate and multivariate Cox regression analyses of the predictors of OS in DPM group.

| Characteristic | Univariate analysis | | Multivariate analysis | |
| --- | --- | --- | --- | --- |
|  | HR [95%CI] | *P* | HR [95%CI] | *P* |
| Age (mean±SD） | 0.983[0.966-1.000] | 0.051 | 0.981[0.959-1.003] | 0.092 |
| BMI (mean±SD） | 1.059[0.995-1.126] | 0.070 | 1.075[1.005-1.151] | 0.037 |
| Gender |  |  |  |  |
| Male | Reference |  |  |  |
| Female | 10.73[0.676-1.702] | 0.766 |  |  |
| Tumor location |  |  |  |  |
| Proximal | Reference |  |  |  |
| Distal | 0.918[0.589-1.432] | 0.707 |  |  |
| Middle | 1.555[0.601-4.022] | 0.362 |  |  |
| Unknown | NA |  |  |  |
| Neoadjuvant therapy |  |  |  |  |
| No | Reference |  |  |  |
| Yes | 1.156[0.557-2.400] | 0.697 |  |  |
| Unknown | 0.865[0.373-2.004] | 0.735 |  |  |
| Gastric stump carcinoma |  |  |  |  |
| No | Reference |  |  |  |
| Yes | 0.741[0.268-2.048] | 0.564 |  |  |
| Unknown | 0.349[0.196-0.622] | <0.001 |  |  |
| Resection type |  |  |  |  |
| PG | Reference |  |  |  |
| DG | 0.894[0.564-1.416] | 0.632 |  |  |
| TG | 1.522[0.768-3.019] | 0.229 |  |  |
| Unknown | 0.653[0.273-1.560] | 0.337 |  |  |
| Surgical approach |  |  |  |  |
| Open | Reference |  |  |  |
| Laproscope | 1.009[0.602-1.693] | 0.972 |  |  |
| Tumor size (pathology) |  |  |  |  |
| <5cm | Reference |  |  |  |
| ≥5cm | 1.369[0.812-2.307] | 0.238 |  |  |
| Unknown | 1.362[0.683-2.718] | 0.381 |  |  |
| Differentiation |  |  |  |  |
| Well and Moderate | Reference |  |  |  |
| Poor and Undifferentiated | 1.711[0.818-3.576] | 0.154 |  |  |
| Unknown | 1.731[0.733-4.090] | 0.211 |  |  |
| Borrman classification |  |  |  |  |
| Ⅰ | Reference |  | Reference |  |
| Ⅱ | 0.372[0.155-0.893] | 0.027 | 0.399[0.155-1.026] | 0.057 |
| Ⅲ | 0.827[0.387-1.769] | 0.624 | 0.596[0.263-1.349] | 0.215 |
| Ⅳ | 0.684[0.291-1.610] | 0.385 | 0.614[0.237-1.593] | 0.316 |
| Unknown | 0.304[0.119-0.774] | 0.012 | 0.469[0.123-1.793] | 0.269 |
| Lauren classification |  |  |  |  |
| intestinal type | Reference |  |  |  |
| diffuse type | 1.660[0.719-3.832] | 0.235 |  |  |
| mixed type | 1.970[0.623-6.218] | 0.248 |  |  |
| Unknown | 1.401[0.635-3.091] | 0.404 |  |  |
| pT stage |  |  |  |  |
| T1 | Reference |  | Reference |  |
| T2 | NA | 0.968 | NA | 0.970 |
| T3 | 4.249[0.975-18.516] | 0.054 | 2.333[0.284-19.152] | 0.430 |
| T4 | 7.285[1.782-29.782] | 0.006 | 4.774[0.633-36.033] | 0.130 |
| Unknown | 7.061[0.636-78.367] | 0.111 | 16.816[1.082-261.235] | 0.044 |
| Number of nodes retrived (mean±SD) | 0.987[0.971-1.003] | 0.100 |  |  |
| pN stage |  |  |  |  |
| N0 | Reference |  | Reference |  |
| N1 | 1.541[0.637-3.725] | 0.337 | 1.199[0.431-3.333] | 0.728 |
| N2 | 2.376[1.118-5.047] | 0.024 | 1.960[0.761-5.052] | 0.163 |
| N3 | 2.572[1.333-4.964] | 0.005 | 1.756[0.715-4.314] | 0.220 |
| Unknown | 3.555[0.784-16.111] | 0.100 | 1.373[0.111-17.004] | 0.805 |
| Lymphatic vessels invasion |  |  |  |  |
| Negative | Reference |  | Reference |  |
| Positive | 1.609[1.023-2.532] | 0.040 | 0.974[0.551-1.722] | 0.928 |
| Unknown | 0.939[0.458-1.923] | 0.863 | 0.389[0.153-0.986] | 0.047 |
| Nerve invasion |  |  |  |  |
| Negative | Reference |  |  |  |
| Positive | 1.387[0.869-2.213] | 0.170 |  |  |
| Unknown | 0.777[0.383-1.575] | 0.484 |  |  |
| Combined resection |  |  |  |  |
| No | Reference |  |  |  |
| Yes | 1.632[0.998-2.668] | 0.051 | 1.261[0.681-2.332] | 0.460 |
| Postoperative complications |  |  |  |  |
| No | Reference |  |  |  |
| Yes | 0.966[0.482-1.938] | 0.923 |  |  |
| Unknown | 0.862[0.456-1.630] | 0.648 |  |  |
| Postoperative treatment |  |  |  |  |
| No | Reference |  |  |  |
| Yes | 1.231[0.623-2.429] | 0.550 | 0.503[0.191-1.324] | 0.164 |
| Chemotherapy | 1.231[0.613-2.472] | 0.559 | 0.801[0.327-1.960] | 0.627 |
| Chemoraidotherapy | 1.284[0.554-2.973] | 0.560 | 0.811[0.294-2.237] | 0.685 |
| Reoperation | 4.454[1.375-14.430] | 0.013 | 4.800[0.999-23.072] | 0.050 |
| Unknown | 1.201[0.581-2.483] | 0.621 | 0.637[0.256-1.583] | 0.331 |

NA: Not Available;

Supplementary Table 7: Univariate and multivariate Cox regression analyses of the predictors of RFS in DPM group.

| Characteristic | Univariate analysis | | Multivariate analysis | |
| --- | --- | --- | --- | --- |
|  | HR [95%CI] | *P* | HR [95%CI] | *P* |
| Age (mean±SD） | 0.970[0.947-0.993] | 0.012 | 0.959[0.929-0.989] | 0.008 |
| BMI (mean±SD） | 0.965[0.882-1.055] | 0.434 |  |  |
| Gender |  |  |  |  |
| Male | Reference |  |  |  |
| Female | 0.963[0.486-1.908] | 0.914 |  |  |
| Tumor location |  |  |  |  |
| Proximal | Reference |  |  |  |
| Distal | 1.258[0.646-2.451] | 0.500 |  |  |
| Middle | 0.666[0.086-5.127] | 0.696 |  |  |
| Unknown | NA |  |  |  |
| Neoadjuvant therapy |  |  |  |  |
| No | Reference |  |  |  |
| Yes | 1.723[0.672-4.420] | 0.257 |  |  |
| Unknown | 2.154[0.896-5.181] | 0.086 |  |  |
| Gastric stump carcinoma |  |  |  |  |
| No | Reference |  |  |  |
| Yes | 2.052[0.720-5.845] | 0.178 |  |  |
| Unknown | 0.707[0.344-1.454] | 0.346 |  |  |
| Resection type |  |  |  |  |
| PG | Reference |  |  |  |
| DG | 1.176[0.583-2.371] | 0.651 |  |  |
| TG | 1.553[0.503-4.788] | 0.444 |  |  |
| Unknown | 2.116[0.842-5.318] | 0.111 |  |  |
| Surgical approach |  |  |  |  |
| Open | Reference |  |  |  |
| Laproscope | 1.330[0.671-2.635] | 0.414 |  |  |
| Tumor size (pathology) |  |  |  |  |
| <5cm | Reference |  |  |  |
| ≥5cm | 1.765[0.768-4.057] | 0.181 |  |  |
| Unknown | 2.531[0.941-6.809] | 0.066 |  |  |
| Differentiation |  |  |  |  |
| Well and Moderate | Reference |  |  |  |
| Poor and Undifferentiated | 1.333[0.465-3.819] | 0.593 |  |  |
| Unknown | 2.842[0.914-8.837] | 0.071 |  |  |
| Borrman classification |  |  |  |  |
| Ⅰ | Reference |  |  |  |
| Ⅱ | 0.762[0.152-3.807] | 0.740 |  |  |
| Ⅲ | 1.296[0.297-5.664] | 0.730 |  |  |
| Ⅳ | 2.203[0.485-9.995] | 0.306 |  |  |
| Unknown | 1.286[0.276-6.001] | 0.749 |  |  |
| Lauren classification |  |  |  |  |
| intestinal type | Reference |  |  |  |
| diffuse type | 0.779[0.260-2.336] | 0.656 |  |  |
| mixed type | NA | 0.978 |  |  |
| Unknown | 1.102[0.427-2.843] | 0.841 |  |  |
| pT stage |  |  |  |  |
| T1 | Reference |  | Reference |  |
| T2 | 1.837[0.115-29.407] | 0.667 | 1.665[0.094-29.564] | 0.728 |
| T3 | 4.835[0.602-38.817] | 0.138 | 7.584[0.781-73.625] | 0.081 |
| T4 | 6.089[0.828-44.759] | 0.076 | 8.594[0.994-74.312] | 0.051 |
| Unknown | 24.172[2.172-268.959] | 0.010 | 18.912[1.213-294.848] | 0.036 |
| Number of nodes retrived (mean±SD) | 0.995[0.974-1.018] | 0.684 |  |  |
| pN stage |  |  |  |  |
| N0 | Reference |  | Reference |  |
| N1 | 1.162[0.413-3.271] | 0.776 | 0.662[0.212-2.069] | 0.478 |
| N2 | 1.043[0.387-2.812] | 0.933 | 0.582[0.186-1.815] | 0.351 |
| N3 | 1.139[0.515-2.519] | 0.749 | 0.406[0.147-1.120] | 0.082 |
| Unknown | 66.336[8.090-543.944] | <0.001 | 15.292[1.519-152.928] | 0.020 |
| Lymphatic vessels invasion |  |  |  |  |
| Negative | Reference |  | Reference |  |
| Positive | 1.925[0.957-3.871] | 0.066 | 2.733[1.082-6.903] | 0.033 |
| Unknown | 1.987[0.810-4.877] | 0.134 | 1.301[0.463-3.659] | 0.618 |
| Nerve invasion |  |  |  |  |
| Negative | Reference |  |  |  |
| Positive | 1.326[0.662-2.655] | 0.426 |  |  |
| Unknown | 1.420[0.610-3.304] | 0.416 |  |  |
| Combined resection |  |  |  |  |
| No | Reference |  |  |  |
| Yes | 1.017[0.451-2.292] | 0.968 |  |  |
| Postoperative complications |  |  |  |  |
| No | Reference |  |  |  |
| Yes | 1.153[0.449-2.964] | 0.767 |  |  |
| Unknown | 1.107[0.464-2.644] | 0.819 |  |  |
| Postoperative treatment |  |  |  |  |
| No | Reference |  | Reference |  |
| Yes | 0.553[0.270-1.133] | 0.105 | 0.322[0.132-0.785] | 0.013 |
| Chemotherapy | 0.527[0.248-1.119] | 0.095 | 0.365[0.144-0.929] | 0.035 |
| Chemoraidotherapy | 0.628[0.231-1.706] | 0.361 | 0.594[0.194-1.813] | 0.360 |
| Reoperation | 1.494[0.326-6.860] | 0.605 | 0.613[0.106-3.537] | 0.585 |
| Unknown | 0.277[0.107-0.714] | 0.008 | 0.210[0.070-0.628] | 0.005 |

NA: Not Available;

Supplementary Table 8: Univariate and multivariate Cox regression analyses of the predictors of OS in BPM group.

| Characteristic | Univariate analysis | | Multivariate analysis | |
| --- | --- | --- | --- | --- |
|  | HR [95%CI] | *P* | HR [95%CI] | *P* |
| Age (mean±SD） | 1.016[0.974-1.060] | 0.456 |  |  |
| BMI (mean±SD） | 0.910[0.782-1.060] | 0.225 |  |  |
| Gender |  |  |  |  |
| Male | Reference |  |  |  |
| Female | 1.457[0.531-3.997] | 0.465 |  |  |
| Tumor location |  |  |  |  |
| Proximal | Reference |  |  |  |
| Distal | 1.138[0.404-3.208] | 0.807 |  |  |
| Middle | 1.236[0.239-6.392] | 0.801 |  |  |
| Unknown | 2.172[0.247-19.077] | 0.484 |  |  |
| Neoadjuvant therapy |  |  |  |  |
| No | Reference |  |  |  |
| Yes | 0.366[0.049-2.752] | 0.329 |  |  |
| Unknown | 1.223[0.355-4.211] | 0.749 |  |  |
| Gastric stump carcinoma |  |  |  |  |
| No | Reference |  |  |  |
| Yes | 0.469[0.062-3.569] | 0.464 |  |  |
| Unknown | 0.689[0.249-1.906] | 0.474 |  |  |
| Resection type |  |  |  |  |
| PG | Reference |  |  |  |
| DG | 0.407[0.105-1.581] | 0.194 |  |  |
| TG | 0.915[0.306-2.737] | 0.873 |  |  |
| Unknown | 1.154[0.360-3.695] | 0.810 |  |  |
| Surgical approach |  |  |  |  |
| Open | Reference |  |  |  |
| Laproscope | 1.393[0.574-3.383] | 0.464 |  |  |
| Tumor size (pathology) |  |  |  |  |
| <5cm | Reference |  |  |  |
| ≥5cm | 1.580[0.427-5.848] | 0.494 |  |  |
| Unknown | 1.888[0.510-6.988] | 0.341 |  |  |
| Differentiation |  |  |  |  |
| Well and Moderate | Reference |  |  |  |
| Poor and Undifferentiated | 6.874[0.872-54.206] | 0.067 |  |  |
| Unknown | 4.130[0.411-41.555] | 0.229 |  |  |
| Borrman classification |  |  |  |  |
| Ⅰ | Reference |  |  |  |
| Ⅱ | NA | 0.932 |  |  |
| Ⅲ | NA | 0.934 |  |  |
| Ⅳ | NA | 0.927 |  |  |
| Unknown | NA | 0.940 |  |  |
| Lauren classification |  |  |  |  |
| intestinal type | Reference |  |  |  |
| diffuse type | NA | 0.925 |  |  |
| mixed type | NA | 0.933 |  |  |
| Unknown | NA | 0.930 |  |  |
| pT stage |  |  |  |  |
| T1 | Reference |  |  |  |
| T2 | NA | 1.000 |  |  |
| T3 | NA | 0.927 |  |  |
| T4 | NA | 0.931 |  |  |
| Unknown | NA | 1.000 |  |  |
| Number of nodes retrived (mean±SD) | 0.998[0.967-1.030] | 0.901 |  |  |
| pN stage |  |  |  |  |
| N0 | Reference |  | Reference |  |
| N1 | 6.625[0.591-74.244] | 0.125 | 10.657[0.650-174.711] | 0.097 |
| N2 | 3.949[0.441-35.373] | 0.220 | 5.285[0.449-62.238] | 0.186 |
| N3 | 8.690[1.131-66.752] | 0.038 | 15.544[1.354-178.394] | 0.028 |
| Unknown | NA | 0.988 | NA | 0.987 |
| Lymphatic vessels invasion |  |  |  |  |
| Negative | Reference |  |  |  |
| Positive | 1.265[0.422-3.793] | 0.675 |  |  |
| Unknown | 1.303[0.413-4.116] | 0.652 |  |  |
| Nerve invasion |  |  |  |  |
| Negative | Reference |  |  |  |
| Positive | 2.101[0.710-6.218] | 0.180 |  |  |
| Unknown | 1.791[0.600-5.351] | 0.296 |  |  |
| Combined resection |  |  |  |  |
| No | Reference |  |  |  |
| Yes | 1.786[0.390-8.185] | 0.455 |  |  |
| Postoperative complications |  |  |  |  |
| No | Reference |  |  |  |
| Yes | 1.825[0.583-5.716] | 0.302 |  |  |
| Unknown | 1.890[0.701-5.095] | 0.209 |  |  |
| Postoperative treatment |  |  |  |  |
| No | Reference |  |  |  |
| Yes | 0.931[0.256-3.395] | 0.914 | 0.972[0.252-3.749] | 0.968 |
| Chemotherapy | 0.867[0.224-3.364] | 0.837 | 0.910[0.228-3.617] | 0.893 |
| Chemoraidotherapy | 1.122[0.225-5.595] | 0.888 | 1.608[0.269-9.614] | 0.602 |
| Reoperation | 0.465[0.048-4.494] | 0.508 | 5.710[0.367-88.879] | 0.213 |
| Unknown | 0.799[0.206-3.100] | 0.745 | 0.941[0.238-3.721] | 0.931 |

NA: Not Available;

Supplementary Table 9: Univariate and multivariate Cox regression analyses of the predictors of RFSS in BPM group.

| Characteristic | | Univariate analysis | | Multivariate analysis | |
| --- | --- | --- | --- | --- | --- |
|  |  | HR [95%CI] | *P* | HR [95%CI] | *P* |
| Age (mean±SD） | | 0.993[0.945-1.043] | 0.768 |  |  |
| BMI (mean±SD） | | 0.873[0.724-1.052] | 0.153 |  |  |
| Gender |  |  |  |  |  |
| Male |  | Reference |  |  |  |
| Female |  | 0.617[0.139-2.747] | 0.526 |  |  |
| Tumor location | |  |  |  |  |
| Proximal |  | Reference |  |  |  |
| Distal |  | 0.779[0.234-2.591] | 0.684 |  |  |
| Middle |  | 1.425[0.260-7.795] | 0.683 |  |  |
| Unknown |  | 31.116[1.725-561.180] | 0.020 |  |  |
| Neoadjuvant therapy | |  |  |  |  |
| No |  | Reference |  |  |  |
| Yes |  | 0.479[0.062-3.693] | 0.480 |  |  |
| Unknown |  | 2.956[0.630-13.863] | 0.169 |  |  |
| Gastric stump carcinoma | | |  |  |  |
| No |  | Reference |  |  |  |
| Yes |  | NA | 0.988 |  |  |
| Unknown |  | 0.295[0.066-1.316] | 0.109 |  |  |
| Resection type | |  |  |  |  |
| PG |  | Reference |  |  |  |
| DG |  | 0.932[0.233-3.736] | 0.921 |  |  |
| TG |  | 1.139[0.284-4.567] | 0.854 |  |  |
| Unknown |  | 2.256[0.497-10.240] | 0.292 |  |  |
| Surgical approach | |  |  |  |  |
| Open |  | Reference |  |  |  |
| Laproscope | | 0.945[0.300-2.979] | 0.923 |  |  |
| Tumor size (pathology) | | |  |  |  |
| <5cm |  | Reference |  |  |  |
| ≥5cm |  | 3.614[0.451-28.944] | 0.226 |  |  |
| Unknown |  | 3.927[0.472-32.656] | 0.206 |  |  |
| Differentiation | |  |  |  |  |
| Well and Moderate | | Reference |  |  |  |
| Poor and Undifferentiated | | 4.810[0.616-37.584] | 0.134 |  |  |
| Unknown |  | 2.192[0.198-24.206] | 0.522 |  |  |
| Borrman classification | |  |  |  |  |
| Ⅰ |  | Reference |  |  |  |
| Ⅱ |  | NA | 0.939 |  |  |
| Ⅲ |  | NA | 0.949 |  |  |
| Ⅳ |  | NA | 0.933 |  |  |
| Unknown |  | NA | 0.947 |  |  |
| Lauren classification | |  |  |  |  |
| intestinal type | | Reference |  |  |  |
| diffuse type | | 3.637[0.400-33.083] | 0.252 |  |  |
| mixed type | | NA | 0.983 |  |  |
| Unknown |  | 1.540[0.195-12.193] | 0.682 |  |  |
| pT stage |  |  |  |  |  |
| T1 |  | Reference |  |  |  |
| T2 |  | NA | 1.000 |  |  |
| T3 |  | NA | 0.959 |  |  |
| T4 |  | NA | 0.958 |  |  |
| Unknown |  | NA |  |  |  |
| Number of nodes retrived (mean±SD) | | 0.974[0.930-1.019] | 0.250 |  |  |
| pN stage |  |  |  |  |  |
| N0 |  | Reference |  |  |  |
| N1 |  | NA | 0.937 |  |  |
| N2 |  | NA | 0.937 |  |  |
| N3 |  | NA | 0.933 |  |  |
| Unknown |  | NA |  |  |  |
| Lymphatic vessels invasion | | |  |  |  |
| Negative |  | Reference |  |  |  |
| Positive |  | 1.857[0.476-7.172] | 0.375 |  |  |
| Unknown |  | 2.192[0.521-9.233] | 0.285 |  |  |
| Nerve invasion | |  |  |  |  |
| Negative |  | Reference |  |  |  |
| Positive |  | 1.103[0.309-3.930] | 0.880 |  |  |
| Unknown |  | 1.808[0.549-5.953] | 0.330 |  |  |
| Combined resection | |  |  |  |  |
| No |  | Reference |  |  |  |
| Yes |  | 3.608[0.951-13.683] | 0.059 |  |  |
| Postoperative complications | | |  |  |  |
| No |  | Reference |  |  |  |
| Yes |  | 1.303[0.336-5.058] | 0.702 |  |  |
| Unknown |  | 1.596[0.506-5.045] | 0.425 |  |  |
| Postoperative treatment | | |  |  |  |
| No |  | Reference |  |  |  |
| Yes |  | 0.964[0.199-4.662] | 0.964 |  |  |
| Chemotherapy | | 0.675[0.123-3.705] | 0.651 |  |  |
| Chemoraidotherapy | | 2.234[0.369-13.510] | 0.381 |  |  |
| Reoperation | | NA | 0.988 |  |  |
| Unknown |  | 1.045[0.210-5.207] | 0.957 |  |  |

NA: Not Available;
